# Primary Urethral Malignant Peripheral Neural Sheath Tumor in a 58-Year-Old Female in the Absence of Neurofibromatosis Type 1

**DOI:** 10.7759/cureus.32634

**Published:** 2022-12-17

**Authors:** Mikaela N Brentlinger, Osvaldo Padilla, Jesse Qiao

**Affiliations:** 1 Pathology, Texas Tech University Health Sciences Center Paul L. Foster School of Medicine, El Paso, USA

**Keywords:** soft tissue neoplasm, sarcoma soft tissue, malignant peripheral nerve sheath tumor (mpnst), neurofibromatosis 1 (nf1), bladder

## Abstract

A malignant peripheral neural sheath tumor (MPNST) is a malignant soft tissue neoplasm with cellular origin arising from the outer lining of peripheral nerves. Approximately 10 cases have been identified to date where the lower urinary tract was affected. We discuss the case of a female patient that presented with primary MPNST that arose in the urethral tract in the absence of neurofibromatosis type 1 (NF1) or prior malignancies. This patient presented with pain and acute urinary tract symptoms secondary to urethral obstruction by a protruding vaginal mass. The patient underwent an incomplete initial resection to alleviate symptoms and to obtain a tissue diagnosis. Three months after the first hospitalization, the patient was re-hospitalized due to the recurrence of symptoms and subsequently underwent a complete tumor excision. The initial resection showed a 7.0 x 4.5 x 4.5 cm aggregate of tan-red to gray tumor masses. Microscopic examination showed a spindle cell neoplasm with malignant cytological features (hypercellularity, atypical mitoses, nuclear pleomorphism, and indistinct borders). Tumor cells stained positive for SOX10, S-100 (10% of tumor), with a “mosaic pattern” of H3K27ME3 (50% of tumor nuclei positive). Other lineage-specific and keratin markers stained negative.

In the absence of other patient known primaries, the findings were consistent with a primary MPNST of the urinary tract. Residual tumor was identified on MRI scans one month after the follow-up. The completely excised tumor specimen on the second admission showed identical morphology when compared to the first specimen. While MPNSTs typically carry a poor prognosis, knowledge of behavior and prognosis of primary MPNSTs in the bladder is limited, due to the few relative numbers of available case reports. Further research is needed to study the clinical behavior, morphology, immunophenotypes, and genetics of primary MPNSTs arising from the lower urinary tract.

## Introduction

Malignant peripheral neural sheath tumors (MPNST) are sarcomas that typically arise from the outer lining of peripheral nerves, and typically present as a mass adjacent to or near a peripheral nerve [1]. Approximately one-half of all MPNSTs are sporadic [2-3] and usually present in middle-aged individuals [4]. The other half of MPNSTs occur in younger patients [4] with neurofibromatosis type 1 (NF1) [2-3], inherited in an autosomal dominant condition. While sporadic MPNSTs equally affect men and women [5], NF1-associated MPNSTs are more common in men [2]. In sporadic cases, prior radiation exposure is an established risk factor [6]. MPNSTs typically carry a poor prognosis, highlighting the need for accurate diagnosis and the relevance of additional studies and treatment.

MPNST affecting the lower urinary tract is rare. To date, 10 cases have been described in the literature, all of which were male [7-17]. A total of five patients did not present with a history of NF1 [7-11]. Nine cases reported urinary bladder involvement [7,9-10,12-17], with six cases occurring in the setting of NF1 [12-17]. One case reported urethral involvement in a 14-month-old male, with no history of NF1, that spanned the entire length of the penile urethra [8], requiring radical resection and reconstructive surgery at follow-up [10]. In this case report, we describe a case of primary urethral MPNST in a 58-year-old female.

## Case presentation

We report the case of a 58-year-old female, gravida 4, para 4, who presented to the emergency department for worsening pelvic discomfort with urinary obstruction in the last several days. She was seen by her primary care nurse practitioner three to four weeks ago for pelvic pain during which a pelvic exam showed a prune-sized mass. While the patient was awaiting a specialist referral, her pain worsened as the mass rapidly enlarged. In addition, the patient experienced new-onset minor spotting. She had no known personal or family history of NF1 or cancer. She denied tobacco, alcohol, or illicit drug use. Her age-appropriate cancer screenings were negative to date. Her surgical history was significant for a total abdominal hysterectomy for leiomyomas (fibroids) with mesh abdominal hernia repair.

The inpatient gynecology-oncology team performed a physical exam that was significant for a protruding mass that arose from the vaginal tract with red discoloration, which was not initially identified on the triaging CT scan. The patient underwent subsequent exploratory laparotomy with partial resection of the mass. She was subsequently discharged without complications for outpatient follow-up.

The patient had a scheduled MRI performed one month after the previous visit. She had not yet received radiation or chemotherapy. Three months after her first admission, the patient returned to the hospital for worsening pelvic pain. During the second admission, she underwent complete tumor excision by the gynecology-oncology team and was uneventfully discharged. Subsequent patient follow-up was not available for review.

**Figure 1:**
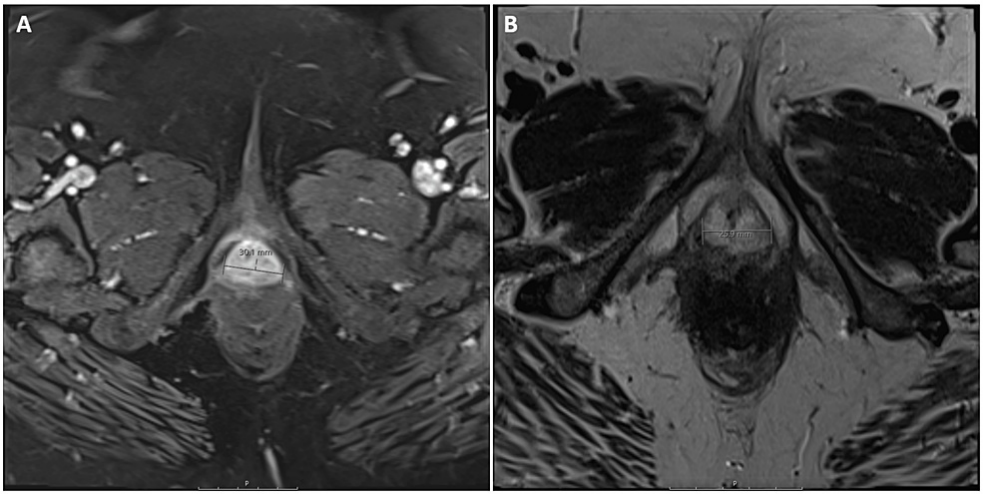
MRI findings MRI of the pelvis, T1-weighted (A) and T2-weighted (B), demonstrated a residual mass at the urethral meatus, performed approximately three weeks after initial resection.

An abdominal/pelvic CT scan with contrast from the patient’s first admission was only significant for a small ventral abdominal hernia without incarceration; a mass was not visualized. The patient’s follow-up pelvic MRI, three weeks following discharge, showed a 30.1 mm hyperintense lobulated mass at the external urethral meatus, visualized on both T1 and T2 enhanced phases. No associated lymphadenopathy, bone lesions, or additional soft tissue lesions were seen. The MRI findings are shown in Figure 1.

The urethral tumor specimen from the patient’s first surgery was received in multiple, irregular fragments that measured 7.0 x 4.5 x 3.5 cm in aggregate, weighing 62 grams. The tumor was tan-red with focal areas of green-to-gray discoloration. The tumor also had gross areas of myxoid changes and friable cut surfaces, which were suggestive of necrosis.

By morphology, the tumor showed a spindle cell, soft tissue neoplasm with high cellularity on low power. High-power examination showed malignant features that consisted of moderate to marked cytological atypia, nuclear pleomorphism, and scattered atypical mitoses (some of which were also recognizable on low- and medium-power magnifications). Approximately one-third of the sampled tumor showed coagulative-type necrosis and inflammation. No surrounding urinary bladder muscle or nerve tissue wall was identified microscopically.

**Figure 2:**
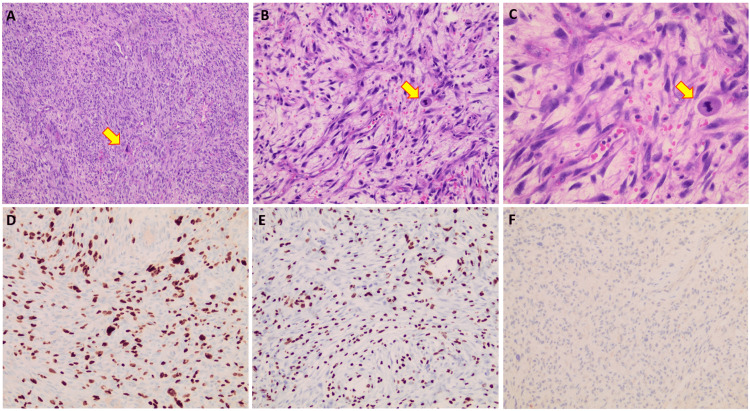
Representative microscopic findings Hematoxylin and eosin sections of the tumor at low power (A, 100x magnification), medium power (B, 200x magnification), and high power (C, 400x magnification) showed a spindle cell neoplasm with high grade nuclear features, pleomorphism, and scattered atypical mitoses that could be visualized on both low and high magnifications (yellow arrows). The tumor showed nuclear positivity for SOX10 in approximately 40% of cells (D, 200x magnification) and H3K27me3 in approximately 50% of cells (E, 200x magnification). The tumor was negative for p16 (F, 200x magnification).

Immunohistochemistry showed a tumor that stained positive for S-100 (10% of cells with weak staining) and SOX10 (40% of cells with strong nuclear staining). The tumor was negative for pan-keratin, CD117, DOG1, CD31, desmin, HMB45, MART1, STAT6, SMA, and myogenin markers. Myosin and CAM5.2 both showed nonspecific patchy staining in less than 5% of the tumor, which was not contributory to the final diagnosis. H3K27me3 showed a partial loss in nuclear positivity that affected approximately 50% of the tumor cells. The tumor stained completely negative for p16. Based on tissue morphology, immunohistochemical staining patterns, and exclusion of other entities, a diagnosis of MPNST was made. Highlights of our microscopic findings are presented in Figure 2.

The peri-urethral tumor specimen from the patient’s complete tumor excision, three months later, was also received in fragments, aggregating to 7.0 x 6.0 x 4.0 cm and weighing 77 grams. The diagnosis of MPNST was made by morphological comparison with the initial surgical specimen, and was found to be identical.

## Discussion

Diagnosing MPNST, especially when not associated with NF1, is challenging since there are no established sets of immunohistochemical markers or recurrent patterns of genetic abnormalities that are seen with other soft tissue neoplasms. In addition, the presence of high-grade or undifferentiated features further adds to the diagnostic challenge of differentiating MPNST by morphology. Thus, the diagnosis of MPNST is often based on clinical presentation, morphology, and immunohistochemical staining patterns that favor MPNST while excluding other entities. Our patient’s MPNST demonstrated a locally-aggressive clinical behavior, from the time of initial presentation to initial resection (several weeks), and the time from interval imaging follow-up (tumor size, 3 cm on MRI) to subsequent completion of excision/debulking (the tumor was up to 7 cm on gross examination).

Microscopic differential diagnoses for MPNST include (but not limited to) atypical neurofibroma, cellular schwannoma, monophasic synovial sarcoma, liposarcoma with de-differentiation, spindle cell melanoma, leiomyosarcoma, and low-grade fibromyxoid sarcoma. In our patient’s differential diagnosis workup, the presence of high-grade morphology features (in particular, tumor necrosis in the absence of neoadjuvant therapy) rendered any low-grade diagnoses highly unlikely. Given the volume of tumor submitted and the lack of a retroperitoneal mass, the absence of lipoblasts made a de-differentiated liposarcoma highly unlikely. The morphology and immunohistochemical patterns did not support a schwannoma. Negative lineage-specific markers rendered the diagnosis of leiomyosarcoma (desmin and SMA negative) and synovial sarcoma (pan-keratin and EMA negative) highly unlikely. The weak S-100 and partial SOX10 expression in our case rendered a spindle cell melanoma an unlikely possibility [18].

In the 10 cases of lower urinary tract primary MPNST, complete or patchy S100 positivity was most consistently noted. While either S-100 and SOX10 can often be positive or partially positive in MPNST, these markers are often not specific and can be seen in other tumors with neural crest origins. Table 1 lists the 10 case reports that we reviewed; the last row shows our case.

**Table 1:** Review of case reports that describes primary MPNSTs occurring in the lower urinary tract MPNST, malignant peripheral neural sheath tumor; NF, neurofibromatosis; NF1, neurofibromatosis, type 1 gene; mutation; IHCs, immunohistochemical stains; Gross, pathology specimens for gross evaluation; MRI, magnetic resonance imaging; CT, computed tomography scan; pan-CK, pan-cytokeratin; EMA, epithelial membrane antigen; MSA, muscle specific actin; SMA, smooth muscle actin; NSE, neuron-specific enolase; HMB45, human melanoma black 45; ALK, anaplastic lymphoma kinase; CD, cluster density; CK, cytokeratin; DOG1, discovered on GIST1; MAK6, serine/threonine protein kinase 6; MIB1, mindbomb E3 ubiquitin protein ligase 1; STAT6, signal transducer and activator of transcription 6; Mart1, melanoma antigen recognized by T cells.

Citation	Age (years)	Gender/Race (if known)	History of NF?	Location	Greatest dimensions	Positive IHCs	Negative IHCs
Sari et al.	53	male	none	Anterior urinary bladder wall	Ultrasound: 3.0 cm Gross: 4.0 cm	Not reported	SMA, desmin, neurofilament, CD57, CK7, Pan-CK
Parekh et al., Agard et al.	1 (14 months)	male	none	Entire penile base and urethra	MRI: 5.4 cm	S-100, vimentin	Not reported
Mimata et al.	67	male	none	12 o’clock of urinary bladder neck	Ultrasound: 2.0 cm	Positive: S-100 Negative: Pan-CK, SMA, EMA	
Petracco et al.	50	male	NF1+	Urinary bladder wall	Diffuse involvement, no discrete tumor on imaging	S100, p16, neurofilament, CD56	Pan-CK, EMA, MDM2, p63, ALK, CD117, DOG1, myogenin, desmin, SMA
Dahm et al.	33	Male/ Caucasian	NF1+	Dorsal bladder wall and proximal prostatic urethra	Pelvic CT: 10.3 cm	Not reported	Not reported
Ross et al.	54	male	NF1+	Posterior-superior midline of urinary bladder	Gross: 3.0 cm	Not reported	Not reported
Hulse et al.	52	Male/Caucasian	NF1+	Urinary bladder, not specified	Not reported	Not reported	Not reported
Eltoum et al.	38	Male/Caucasian	none	Urinary bladder trigone, extending through the muscularis propria	Gross: 2.5 cm	S100, vimentin, NSE, MIB1	Pan-CK, MAK6, HMB45, EMA, MSA, desmin, Leu-7, CD1a, CD30, neurofilament, chromogranin
Kalafatis et al.	57	male	NF1+	Left posterior wall of urinary bladder and left perivesicular fat	Gross: Posterior wall: 7.0 cm Perivesicular: 3.5 cm	S100, vimentin, NSE	EMA, SMA, desmin, Pan-CK
							Rober et al.	29	Male/African American	NF1+	Posterior wall of urinary bladder with smaller lesion within the obturator fossa	Gross: 20.0 x 12.0 x 9.0 cm	Vimentin, S100	desmin, myoglobin, Pan-CK, EMA
This case	58	Female	None	Urethral meatus	Gross: 7.0 x 6.0 x 4.0 cm	SOX10, H3K27ME3 (partial), S-100 (10%)	Pan-CK, HMB45, CD117, DOG1, CD31, desmin, Mart1, STAT6, SMA, myogenin

The partial loss of H3K27me3 staining and a negative p16 staining, that was seen in our case, supported our diagnosis of MPNST. Although the genetic basis of MPNST is complex and not attributed to single or recurrent genetic abnormalities, mutations in the PTEN and PIK3CA have been described [3]. In addition, the use of H3K27me3 immunostaining as a surrogate marker to assess for loss of trimethylation at lysine 27 of histone-H3 has been studied on MPNSTs [19]. While a small percentage of MPNSTs demonstrate a complete loss of nuclear staining, the majority of the cases demonstrate partial loss (between 10% to 90%, or a “mosaic pattern”) of H3K27me3 staining, as seen in our case. In MPNSTs with partial loss of H3K27me3 staining, fluorescent-in-situ hybridization may show deletions of either p16, NF1, or both [20].

Similar to the clinical management of other soft tissue sarcomas, the clinical management of MPNST is dependent on the clinical stage, organs involved, and interdisciplinary approaches to care [21]. MPNSTs are clinically aggressive soft tissue sarcomas that are typically managed by resection. While MPNSTs that are not fully resectable or present with metastases were generally deemed incurable, recent clinical trials and immunotherapy regimens that target receptors (including mTOR, MEK, BET, PD-L1, and PDGFR) for other soft tissue sarcomas have shown limited success in disease management [22]. Nonetheless, therapeutic advances in MPNST management are hindered by the lack of consistent and comprehensive genomic data.

## Conclusions

To the best of our knowledge, this was the first case in the literature of a primary urethral MPNST in a female without NF1 or previous radiation treatment. The addition of our case to the current literature brought the male-to-female ratio to 10:1. However, the male gender predilection for urinary tract MPNST that we observed may not be statistically significant, given the low number of cases analyzed to date. It has been suggested that gender-specific sex hormones may contribute toward the growth of Schwann cells, which were thought to give rise to precursor lesions that transform into MPNSTs. Additional case reporting with larger study series may reveal any gender dominance or molecular abnormalities specifically related to primary MPNSTs arising from the urinary tract, which may guide targeted and specific therapeutic options.
